# Semi-supervised Counting of Grape Berries in the Field Based on Density Mutual Exclusion

**DOI:** 10.34133/plantphenomics.0115

**Published:** 2023-11-28

**Authors:** Yanan Li, Yuling Tang, Yifei Liu, Dingrun Zheng

**Affiliations:** ^1^ School of Computer Science and Engineering, School of Artificial Intelligence, Wuhan Institute of Technology, Wuhan 430205, China.; ^2^ Hubei Key Laboratory of Intelligent Robot, Wuhan Institute of Technology, Wuhan 430073, China.

## Abstract

Automated counting of grape berries has become one of the most important tasks in grape yield prediction. However, dense distribution of berries and the severe occlusion between berries bring great challenges to counting algorithm based on deep learning. The collection of data required for model training is also a tedious and expensive work. To address these issues and cost-effectively count grape berries, a semi-supervised counting of grape berries in the field based on density mutual exclusion (CDMENet) is proposed. The algorithm uses VGG16 as the backbone to extract image features. Auxiliary tasks based on density mutual exclusion are introduced. The tasks exploit the spatial distribution pattern of grape berries in density levels to make full use of unlabeled data. In addition, a density difference loss is designed. The feature representation is enhanced by amplifying the difference of features between different density levels. The experimental results on the field grape berry dataset show that CDMENet achieves less counting errors. Compared with the state of the arts, coefficient of determination (*R*^2^) is improved by 6.10%, and mean absolute error and root mean square error are reduced by 49.36% and 54.08%, respectively. The code is available at https://github.com/youth-tang/CDMENet-main.

## Introduction

Grape is one of the most widely cultivated economic fruits in the world. Yield estimation plays a very important role in optimizing the quality management and economic efficiency of vineyards [1–3]. Traditionally, yield estimation is obtained by manually counting the number of grape clusters. However, this method offers limited accuracy and effectiveness of results due to its high destruction, subjectivity, and cost [4,5]. Nowadays, some researchers have demonstrated the positive impact and crucial role of berry number in yield estimation, as it contributes to obtaining more accurate results [6–8]. With increasing labor costs and the development of modern agriculture, automated berry counting has become a vital task [9]. Therefore, a simple and efficient automatic counting method for grape berries in the field is necessary, which provides useful information for yield prediction.

Driven by computer vision and deep learning, object counting algorithms based on convolutional neural networks have brought new possibilities. Most existing studies utilize detection-based [10–13] and density estimation-based [14–16] approaches to count crops. The detection-based methods detect objects and count their number. However, the variability of farmland can pose substantial challenges to the robustness of models. Although the above methods can detect areas of the objects through bounding boxes, they are more specialized in detecting individual objects. For densely distributed and heavily occluded objects such as grape berries, high counting errors still occur. The density estimation-based methods have been widely applied to object counting, such as crowd counting [17–21], traffic flow monitoring [22], animal and plant quantity statistics [16,23,24], etc. These methods are supervised using point labels and obtain the number of objects by summing up the integrals of the density map. Point labeling can accurately represent the position of objects in dense scenes to reduce counting errors. However, high-performance algorithms in deep learning often rely on large amounts of annotation [25–27]. Acquiring the accurate data is an expensive and time-consuming work actually [28]. High-cost manual annotation is also one of the major reasons limiting the generalization of deep learning models.

Based on aforementioned issues, a semi-supervised counting of grape berries in the field based on density mutual exclusion (CDMENet) is proposed. The method addresses the problem of berry counting for large-scale cultivation with low annotation costs. Unlike previous semi-supervised methods, auxiliary tasks based on density mutual exclusion are introduced. While the model learns from unlabeled images, auxiliary tasks redirect the focus away from density regressor, mitigating the impact of noise. The multiple binary segmentation predictors in these tasks enable feature extractor to emphasize valuable feature learning. Furthermore, spatial contextual information is essential for distinguishing between object and background. Segmentation tasks learn spatial information for objects at different density levels. These allow the density regressor to concentrate on regions of interest, thereby enhancing counting performance. Finally, a density difference loss is designed to enhance feature representation by expanding the feature difference between density levels.

In summary, this paper has the following contributions:

• A semi-supervised counting grape berries in the field based on density mutual exclusion is proposed to solve the statistical problem of grape berries in large-scale cultivation. Compared with the state of the arts, *R*^2^ is improved by 6.10%, mean absolute error (MAE) and root mean square error (RMSE) are reduced by 49.36% and 54.08%, respectively.

• Auxiliary tasks based on density mutual exclusion are introduced. By exploiting the spatial distribution pattern of unlabeled images, the performance of the counting model is improved and burden of manual annotation is reduced.

• A density difference loss is designed. By expanding the feature differences between different density levels, feature extractor learns more important feature representations between different densities.

### Related Work

Object counting is a fundamental task in computer vision that aims to estimate the number of objects. This section reviews fully supervised learning and semi-supervised learning in object counting.

#### Object counting

Object counting is roughly divided into 3 methods: detection-based, regression-based, and density estimation-based.

The detection-based approaches aim to construct detection model which can predict the number of bounding boxes of objects in the image. Rong et al. [12] computed tomato clusters with a modified YOLOv5. Zhang et al. [29] obtained the number of multiple fruits in the orchard by YOLOX. However, the detection-based approaches rely more on detecting individual objects and requires additional bounding box annotations, which does not produce satisfactory counting results in crowded scenes.

The regression-based methods establish a mapping relationship between extracted features and number of objects. Idrees et al. [30] performed a scale-invariant feature transform on the head texture features to estimate the crowd size. TeHran et al. [31] calculated the number of wheat spikes using linear iterative clustering. This mapping relationship solves the counting problem of dense objects to a certain extent. However, these methods ignore the spatial distribution information among objects, which affects the counting accuracy.

The density estimation-based methods have played an important role in solving object counting. In agriculture, Lu et al. [14] proposed a local regression model based on convolutional neural network, to obtain information for corn kernels, wheat ears, and sorghum heads. Bai et al. [32] compiled rice position and size information into decoding network to achieve more accurate rice plant numbers. In the field of crowd counting, Li et al. [33] used dilated convolution to aggregate multiscale context information in crowd scenes. Liu et al. [34] performed feature extraction across multiple receptive fields to optimize essential features. Wang et al. [19] designed a more efficient encoding-decoding network structure, utilizing multilayer feature fusion to further expand feature information. However, these density estimation-based models often rely on a large amount of accurate labeled data, which is expensive and time-consuming.

#### Semi-supervised learning

Object counting based on semi-supervised learning attempts to improve performance with more unlabeled data at a lower cost of annotation. Xu et al. [35] considered existence of similar regions in dense images and employ partial annotations from each image as training data. However, these approaches generally require the selection of regions. Liu et al. [36,37] established a ranking strategy among unlabeled images to estimate the number of objects more accurately. Gao et al. [38] proposed a coarse-to-fine feature margin ranking loss for model training. Liu et al. [39] obtained more robust feature extractors through ranking density among unlabeled images. The ranking constraints of these above methods are almost always satisfied, providing relatively less supervision on unlabeled data [40]. Peng et al. [41] introduced an agent task based on the sum of patch counts in the same unlabeled image. However, when unlabeled data is directly applied to the density regression [36,38,41], the large amount of noise makes training a robust density regression more difficult [39].

To address the aforementioned issues and further improve counting performance in CDMENet, auxiliary tasks have been introduced. These tasks help reduce noise from unlabeled images to some extent. During the training of density regression models, a substantial amount of inherent noise supervision is generated from unlabeled images, which negatively impacts the performance of the model. These tasks redirect the focus of unlabeled images from the density regressor, reducing the interference of noise. Additionally, feature extraction plays a crucial role in prediction. Feature extractor is able to concentrate on learning useful features by employing multiple binary segmentation predictors. More importantly, previous counting methods have demonstrated that spatial region information from binary segmentation tasks is crucial for distinguishing object and background [42–44]. Auxiliary tasks utilize the spatial information of the object at different density levels, which benefits the density regressor in focusing on regions of interest.

## Materials and Methods

### Data acquisition

**Fig. 1.**
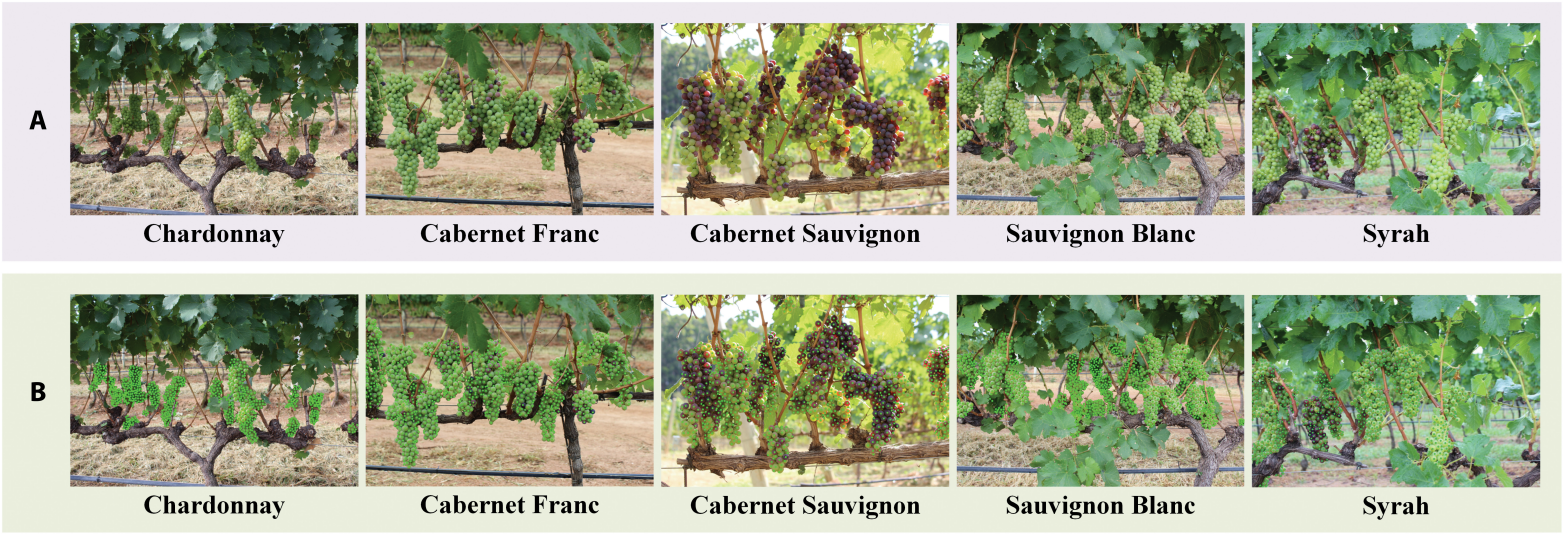
(A and B) Examples of grape species and manually labeled images in the field grape berry dataset.

Embrapa Wine Grape Instance Segmentation Dataset [45] can be well used for grape berry counting by studying the image quality and annotation feasibility. Therefore, 300 images were selected to form the field grape berry dataset in this paper, including 250 images of 5, 184 × 3, 456 pixels and 50 images of 1, 296 × 864 pixels. The field grape berry dataset was divided into 5 grape varieties as shown in Fig. 1A, termed Chardonnay, Cabernet Franc, Cabernet Sauvignon, Sauvignon Blanc, and Syrah.

The “Labelme” annotation tool was used for labeling the dataset. Grape berries in each image were manually labeled to obtain the ground truth, and an example of manual annotation is shown in Fig. 1B. The number of grape berries ranged from 206 to 1,585 in each image, and a total of 187,372 berries were labeled in 300 images. Annotation files and the images were randomly assigned to training, validation, and test sets in a 3:1:1 ratio.

### Methods

**Fig. 2.**
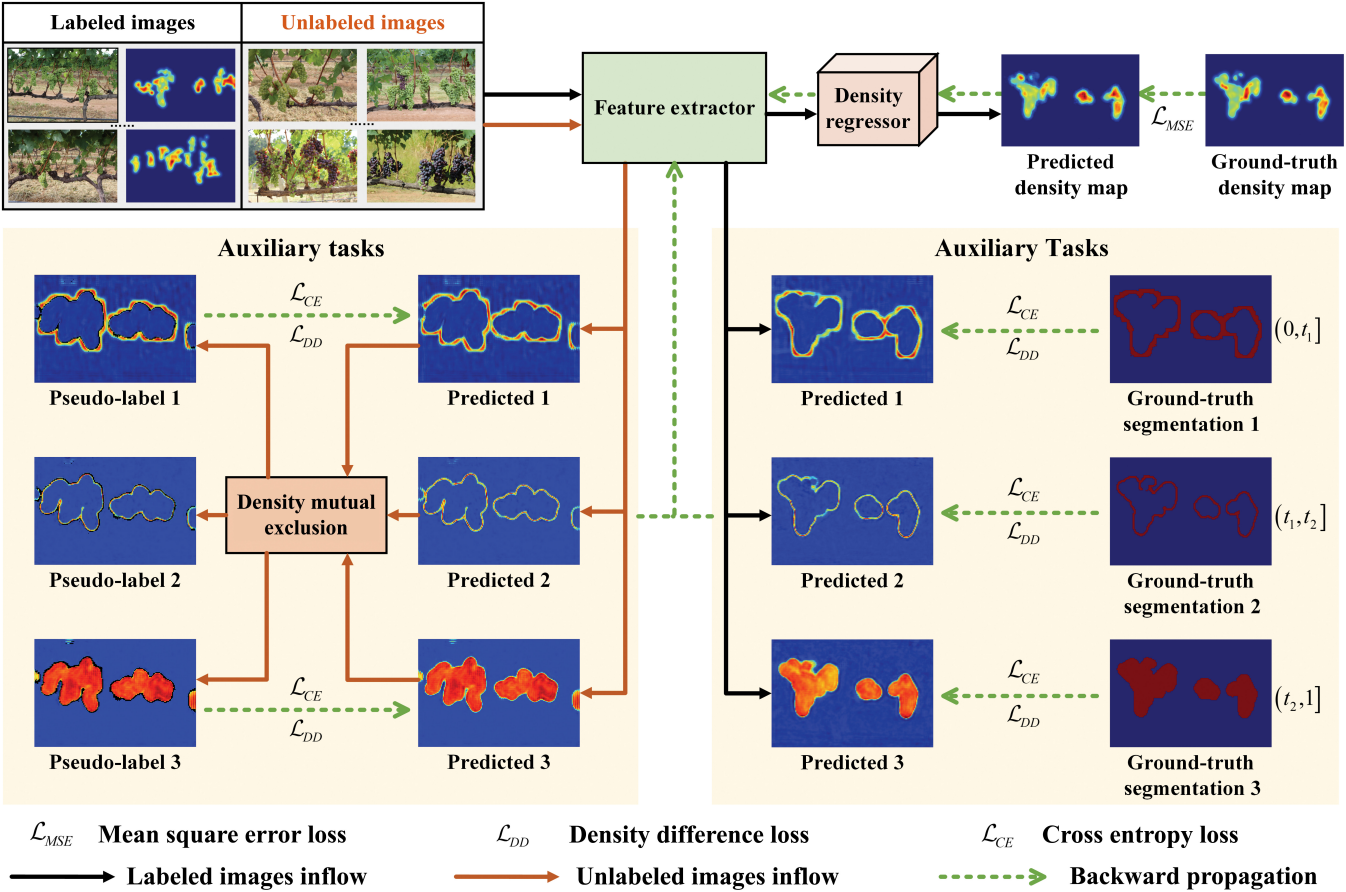
A semi-supervised counting framework of grape berries in the field based on density mutual exclusion. Unlabeled images only flow into the feature extractor and auxiliary tasks.

The framework of the proposed CDMENet is shown in Fig. 2. CDMENet consists of a feature extractor, a density regressor, and auxiliary tasks. The feature extractor extracts the deep features. The density regressor predicts the density map of berries. The auxiliary tasks provide CDMENet with spatial distribution of objects across density levels and redirect the focus of unlabeled images to enhance model performance. In addition, a density difference loss is designed to enhance the feature representation of the feature extractor at different density levels.

#### Settings required for semi-supervised counting

Semi-supervised counting requires a set of labeled images $D_{l}={\left\{I_{i}^{l},D_{i}\right\}}_{i=1}^{N_{l}}$ and a set of unlabeled images $D_{u}={\left\{I_{i}^{u}\right\}}_{i=1}^{N_{u}}$, where $I_{i}^{l}$ is labeled image, *D_i_* is the corresponding ground-truth density map, *N_l_* is the number of labeled images, $I_{i}^{u}$ is an unlabeled image without a corresponding ground-truth density map, and *N_u_* is the number of unlabeled images. In semi-supervised counting, the number of unlabeled images is much greater than the number of labeled images, i.e., *N_u_* ≫ *N_l_*.

Density map estimation is equivalent to calculate the density values of all pixels in the map, and the sum of the density maps is equal to the number of objects in the map. In this paper, a fixed size Gaussian kernel is used to blur the annotations of each grape berry to generate ground-truth density maps. The specific calculation is as follows:

$$D\left(x\right)=\sum_{n=1}^{N}‍\sigma \left(x-x_{n}\right)*G_{\sigma }\left(x\right)$$ (1)

where *x* is the position of each pixel in grape image, *x_n_* is the position of the *n*th annotation point (*N* points in total), and *G_σ_* is a fixed size Gaussian kernel function (*σ* = 15).

#### Feature extraction module

**Fig. 3.**
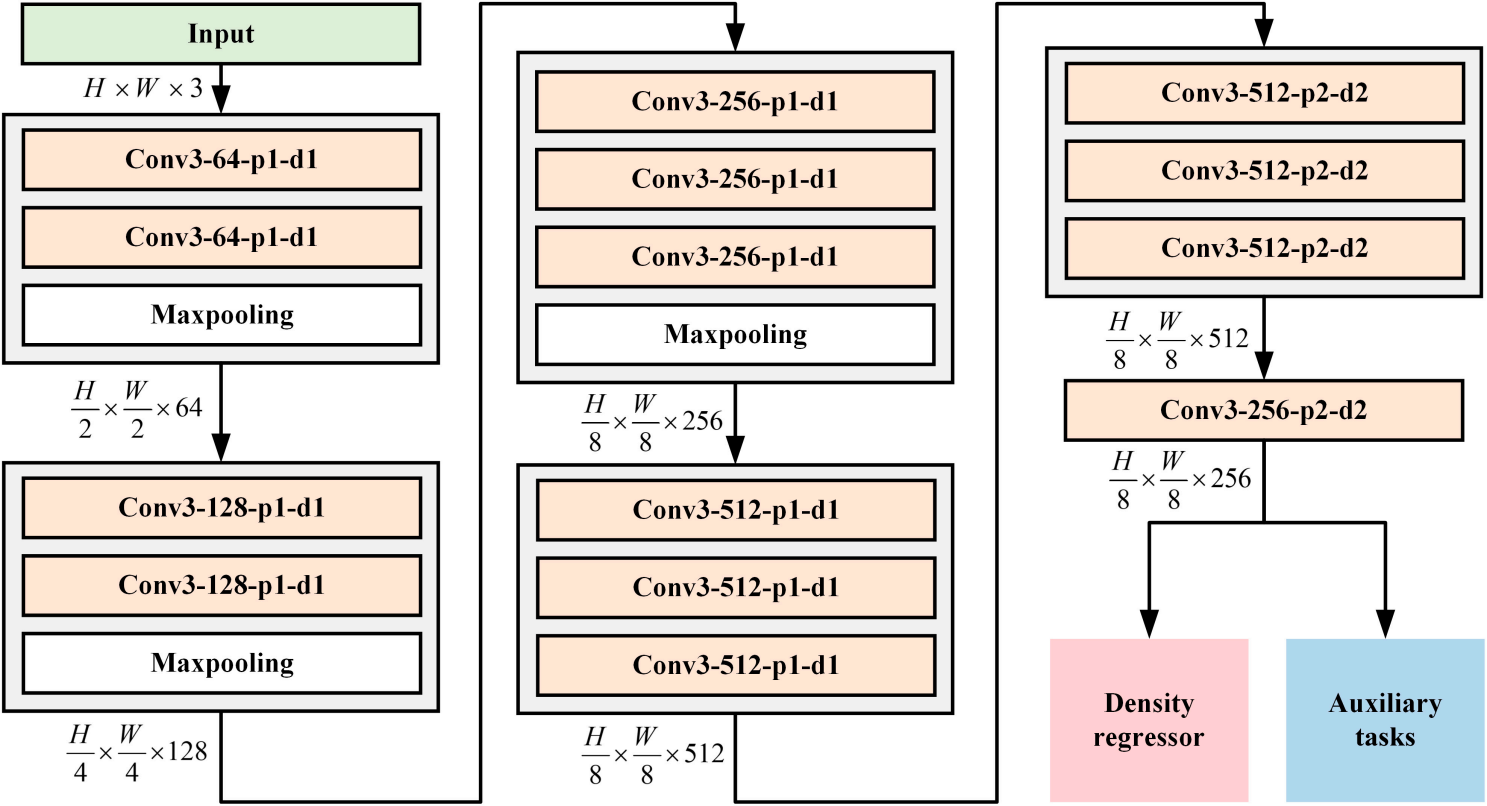
Feature extraction network structure. *Conv*(*n*_1_) − (*n*_2_) − *p*(*n*_3_) − *d*(*n*_4_) is various settings in the convolution operation, *n*_1_ is convolution kernel size, *n*_2_ is the number of output channels, *n*_3_ is the number of padding, and *n*_4_ is the dilated rate.

Inspired by CSRNet [33], the improved feature extractor is adopted in our proposed CDMENet. VGG16 is used as the backbone to extract the deep features of grape berries. The network is shown in Fig. 3. The deep features of the input image (size of *H* × *W* × 3) are extracted by the first 4 convolutional layers to sequentially obtain the feature map of size $\frac{H}{2}\times \frac{W}{2}\times 64$, $\frac{H}{4}\times \frac{W}{4}\times 128$, $\frac{H}{8}\times \frac{W}{8}\times 256$, and $\frac{H}{8}\times \frac{W}{8}\times 512$. In the last 2 layers of the backbone, the feature maps are mapped sequentially into feature maps of size $\frac{H}{8}\times \frac{W}{8}\times 512$ and $\frac{H}{8}\times \frac{W}{8}\times 256$ by dilated convolution with a dilated rate of 2. The final output of the feature extractor flows into 2 modules: density regressor and auxiliary tasks.

#### Auxiliary tasks based on density mutual exclusion

There are 2 critical issues that need to be addressed in semi-supervised object counting. First, there is the challenge of maximizing the utilization of information from unlabeled images. Second, noise from unlabeled images has adverse impact on the density regressor. To address the above problem, the auxiliary tasks are proposed, which serves as follows: (a) The auxiliary tasks redirect the focus of unlabeled images training from the density regressor to feature learning, thus reducing the interference of noise. (b) By training multiple binary segmentation predictors in these tasks, the feature learning capability of CDMENet is improved. (c) These tasks improve model performance by learning the spatial distribution of objects with different density levels, which helps the model to focus more on regions of interest [42–44]. Specifically, the auxiliary tasks consist of multiple density-level predictors. The tasks are designed to predict the spatial distribution of object pixels at different density levels.

The predicted binary segmentation maps $M_{1}^{\prime },M_{2}^{\prime },\ldots ,M_{k}^{\prime }$ are generated by the predictors, which represents the distribution of object pixels at different levels. To obtain accurate distribution predictions, the predictors follow Eq. 2 as the objective for predicting the levels of pixles of different densities and clustering features of the same level. Specifically, it assigns a value of 1 to foreground pixels within the same density threshold and a value of 0 to background pixels outside the threshold:

$$M\left(i,j\right)=\left\{\begin{array}{cc} 1 & t_{1}<D\left(i,j\right)\leq t_{2}\\ 0 & D\left(i,j\right)\geq t_{2}\cup D\left(i,j\right)<t_{1} \end{array}\right.$$ (2)

where (*i*, *j*) is the coordinates of pixels in the image, *M* is the prediction target map, *D* is density map, and *t*_1_ and *t*_2_ are predefined thresholds. To obtain a reasonable threshold range, the nonzero density values in the labeled images are arranged in ascending order to form a sequence {*d_p_*}. The value located at the sequence *S_k_* × *T* is used as the threshold, where *T* is the length of {*d_p_*}, *S_k_* ∈ [0, 1] *k* = 1, 2…, *c*, and *c* is the number of thresholds.

The supervised signal of the labeled images are obtained according to Eq. 2. For unlabeled images with no available supervised signal, CDMENet uses density mutual exclusion relations for supervision. The rules for generating different supervised signals are shown in Algorithm 1. 
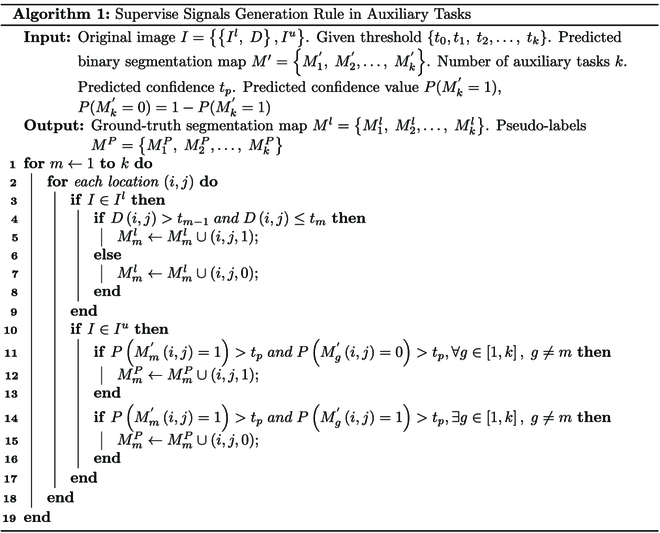


**Fig. 4.**
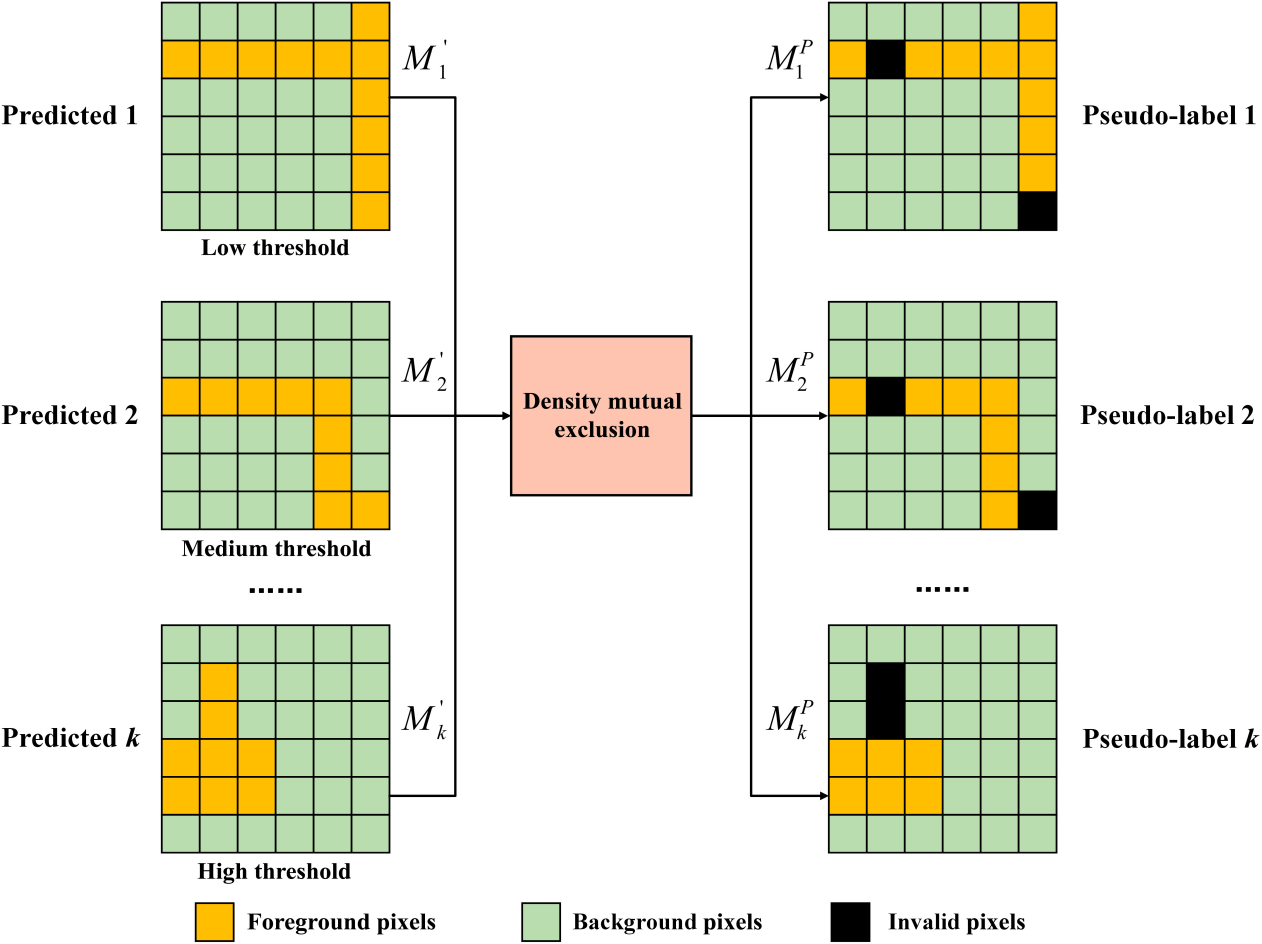
The illustration of pseudo-label generation based on density mutual exclusion.

Density mutual exclusion builds on prior knowledge that the density value of a pixel belongs to only one threshold range. Specifically, the *k* predictions for a pixel (*i*, *j*) are generated by the *k* density level predictors. If the prediction is greater than the prediction confidence *t_p_* = 0.8, the pixel is assigned a foreground label. When multiple predictions of a pixel simultaneously higher than the prediction confidence, it is referred to as a nonmutually exclusive pixel. The spatial information of nonmutually exclusive pixels is considered invalid and is excluded from CDMENet supervision. The pseudo-label *M^P^* is generated by correcting *M*^′^, which is shown in Fig. 4.[Fig F5]

**Fig. 5. F5:**
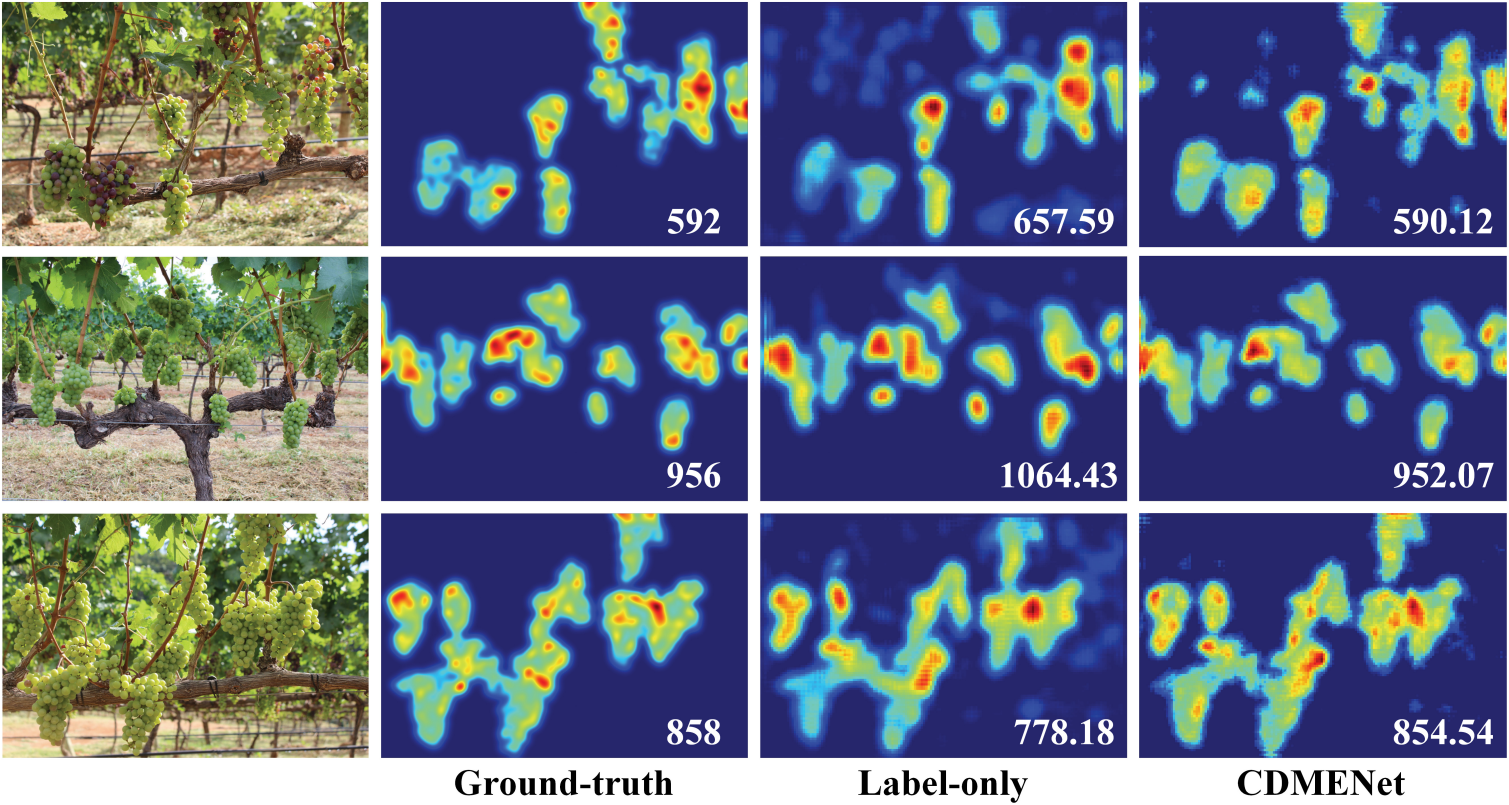
Comparison of predicted density maps on the field grape berry dataset. First column: input images. Second column: ground-truth density maps after Gaussian kernel blur. Third column: predicted density maps trained with only 10% labeled images. Fourth column: predicted density maps trained with 10% labeled images and 90% unlabeled images.

This mutually exclusive relationship serve as an error correction mechanism to remove pixels with contradictory predictions. The generated pseudo-labels take full advantage of the information in the unlabeled images. Cross-entropy loss is used as auxiliary tasks of supervision, defined as:

$$L_{\mathrm{CE}}=-\frac{1}{k}\sum_{i=1}^{k}‍\left(M_{i}^{\prime }\text{log}\left(M_{i}\right)+\left(1-M_{i}^{\prime }\right)\text{log}\left(1-M_{i}\right)\right)$$ (3)

where *k* is the number of auxiliary task predictors, and *M*^′^ is predicted binary segmentation map. For labeled images, *M* is ground-truth segmentation map, and for unlabeled images, *M* is a pseudo-label *M^P^*.

#### Density difference loss

The predictors have a high uncertainty when matching pixels with density values close to the threshold. Incorrect or contradictory predictions have a detrimental effect on model performance. Therefore, a density difference loss is designed to expend the feature differences between density levels. The loss can pull features of the same density level as close as possible and push features of other density levels far away. Density difference loss $L_{\mathrm{DD}}$ is defined as follows:

$$S\left(f_{1},f_{2}\right)=\frac{f_{1}\cdot f_{2}}{\left\|f_{1}\right\|\cdot \left\|f_{2}\right\|}$$ (4)

$$L_{\max}=-\frac{1}{k}\sum_{i=1}^{k}‍\sum_{w=1}^{W}‍S\left(f_{i}^{\prime }\left(w\right),f_{i}\left(w\right)\right)$$ (5)

$$L_{\min}=\frac{1}{k\left(k-1\right)}\sum_{i}^{k}‍\sum_{j}^{k-1}‍\sum_{w=1}^{W}‍S\left(f_{i}^{\prime }\left(w\right),f_{j}\left(w\right)\right)$$ (6)

$$L_{\mathrm{DD}}=L_{\max}+L_{\min}$$ (7)

where *S*(·) is the cosine similarity, *k* is the number of auxiliary task predictors, and *W* is the number of feature vectors. Feature vectors $f_{i}^{\prime }\in M_{i}^{\prime },f_{i}\in M_{i}$ are extracted column by column, and cosine similarity operation is performed for labeled and unlabeled images; *M_i_* is the ground-truth segmentation map and pseudo-label. $L_{\max}$ is adopted to maximize the similarity loss between each predicted map and the corresponding ground-truth map. $L_{\min}$ is employed to minimize the loss of similarity between each predicted map and other irrelevant ground-truth maps.

Finally, the total loss of CDMENet consists of labeled and unlabeled image loss. For labeled images, density regressor and auxiliary tasks construct the loss:

$$L_{\mathrm{MSE}}=\frac{1}{N}\sum_{i=1}^{N}‍{\left\|D_{i}^{\prime }-D_{i}\right\|}_{2}^{2}$$ (8)

$$L_{\text{labeled}}=L_{\mathrm{MSE}}+\lambda_{1}L_{\mathrm{CE}}+\lambda_{2}L_{\mathrm{DD}}$$ (9)

where $L_{\mathrm{MSE}}$ is mean square error loss, *D*^′^ is predicted density map, *D* is ground-truth density map, and *N* is the number of labeled images. $L_{\mathrm{CE}}$ is cross entropy loss. $L_{\mathrm{DD}}$ is density difference loss proposed in the paper. *λ*_1_ and *λ*_2_ are loss parameters, which is 0.01 and 1, respectively.

In CDMENet, unlabeled images are not used to train the density regressor, so the loss $L_{\text{unlabeled}}$ only includes auxiliary tasks:

$$L_{\text{unlabeled}}=\lambda_{3}L_{\mathrm{CE}}+\lambda_{4}L_{\mathrm{DD}}$$ (10)

where *λ*_3_ and *λ*_4_ are loss parameters, which is 0.01 and 1, respectively. The total loss of the whole model is:

$$L_{\text{total}}=L_{\text{labeled}}+L_{\text{unlabeled}}$$ (11)

## Results

### Implement details

The environment for experiment is: Ubuntu 20.04 system, Python 1.7.1 [46] open-source framework, Python 3.8 programming language, Intel Core i9-10900X CPU@3.70GHz, and GeForce RTX 3090. To expand the number of training samples and reduce the computational complexity, the training set images are preprocessed with random cropping (1,296 × 864 pixels) and random flipping during the data loading phase. The values located at {0,0.33,066,1} of sequence {*d_p_*} are sequentially selected as the density thresholds. The network is randomly loaded with different types of images for training, and the batch size is set to 1. The epoch is set to 120. The initial learning rate is 1e-6. The learning rate of the model training will be halved every 30 epochs to ensure that the model converges. Adam algorithm is adopted for optimization to parameters of model [47].

### Evaluation metrics

To validate the effectiveness of proposed CDMENet for counting grape berries in the field, 3 evaluation metrics are used to evaluate the performance of the model, namely MAE, RMSE, and coefficient of determination (*R*^2^). Compared with MAE, RMSE is more sensitive to occurrence of outlier is better suited for evaluating the robustness of the model. *R*^2^ evaluates the degree of fitness between the predicted values of the model and the corresponding ground truth. The specific calculation is as follows:

$$\mathrm{MAE}=\frac{1}{N}\sum_{i-1}^{N}‍\left|C_{i}-C_{i}^{\prime }\right|$$ (12)

$$\text{RMSE}=\sqrt{\frac{1}{N}\sum_{i-1}^{N}‍{\left|C_{i}-C_{i}^{\prime }\right|}^{2}}$$ (13)

$$R^{2}=1-\frac{\sum_{i=1}^{N}‍{\left(C_{i}-C_{i}^{\prime }\right)}^{2}}{\sum_{i=1}^{N}‍{\left(C_{i}-\bar{C}\right)}^{2}}$$ (14)

where *N* is the number of images in the test set. *C_i_* and $C_{i}^{\prime }$ are actual number and predicted number of grape berries in the *i*th image, respectively. $\bar{C}$ is the average number of grape berries.

### Comparison with other counting methods

To validate the effectiveness of CDMENet for field grape berry counting, comparative experiments are conducted in 2 dimensions: fully supervised counting and semi-supervised counting. Fully supervised counting includes CSRNet [33], CANNet [34], MobileCount [19], and TasselNetV2+ [14]. Semi-supervised counting includes Learning to Rank (L2R) [36], IRAST [39], PAL [35], and S2FPR [38]. The abovementioned algorithms are trained separately on the field grape berry dataset. Table 1 shows the values of MAE, RMSE, and *R*^2^ for the test set on different algorithms.

**Table 1.** Experimental results of the field grape berry dataset on various counting algorithms. Note: “Type” is the type of method, i.e., fully supervised algorithm or semi-supervised algorithm. “Ratio” is the ratio of the number of labeled images to the number of train set.

Type	Ratio	Method	MAE	RMSE	*R* ^2^
Fully	100%	CSRNet [33]	192.89	246.03	0.3816
		CANNet [34]	199.73	252.57	0.4547
		MobileCount [19]	45.31	57.32	0.9591
		TasselNetV2+ [14]	41.03	55.00	0.9647
Semi	10%	L2R [36]	83.61	115.79	0.8537
		IRAST [39]	60.50	88.00	0.9222
		PAL [35]	90.97	121.59	0.8167
		S2FPR [38]	—	—	—
		CDMENet(Ours)	30.57	40.21	0.9787
Semi	20%	L2R [36]	72.47	89.86	0.9021
		IRAST [39]	51.52	72.02	0.9389
		PAL [35]	80.37	102.33	0.8503
		S2FPR [38]	102.76	121.77	0.7829
		CDMENet(Ours)	28.01	37.65	0.9817
Semi	30%	L2R [36]	63.81	89.64	0.9148
		IRAST [39]	41.81	60.84	0.9508
		PAL [35]	83.16	106.32	0.8464
		S2FPR [38]	108.42	138.50	0.7753
		CDMENet(Ours)	27.58	37.22	0.9823
Semi	40%	L2R [36]	67.67	94.98	0.8996
		IRAST [39]	44.43	64.51	0.9509
		PAL [35]	85.62	118.13	0.8115
		S2FPR [38]	95.97	135.45	0.8112
		CDMENet(Ours)	27.12	36.12	0.9829
Semi	50%	L2R [36]	60.71	87.99	0.9257
		IRAST [39]	39.11	58.66	0.9612
		PAL [35]	76.04	92.91	0.8868
		S2FPR [38]	90.74	126.71	0.8172
		CDMENet(Ours)	26.47	36.32	0.9839

From Table 1, CDMENet shows the best overall results for grape berry counting in the field. As a semi-supervised method, CDMENet cannot be directly compared with fully supervised methods under the same conditions, but experimental results show that CDMENet can achieve better counting accuracy using only fewer labeled images. In particular, compared with CSRNet and CANNet in full supervision with a ratio of 10%, MAE of CDMENet is reduced by 84.12% and 84.66%, RMSE of CDMENet is reduced by 83.58% and 84.00%, and *R*^2^ of CDMENet is increased by 156.42% and 115.20%, respectively. Similarly, compared to MobileCount and TasselNetV2+, CDMENet also achieves better counting performance with lower annotation costs.

When compared with semi-supervised counting algorithms, CDMENet achieves the less counting error in different ratios. It is worth noting that in the setting of semi-supervised learning, the number of unlabeled images is usually expected to be far more than the number of labeled images. Compared to other algorithms, the performance improvement of CDMENet is more notable when there are fewer labeled images available. For example, when the ratio is 10%, CDMENet improves at least 6.10% in *R*^2^ and reduces at least 49.36% and 54.08% in MAE and RMSE, respectively.

Moreover, the performance improvement of CDMENet shows less pronounced as the number of labeled images increases, further confirming the comprehensive utilization of information from unlabeled images. In summary, the above results demonstrate the effectiveness of CDMENet in field grape berry counting. A comparison of predicted density maps on the dataset is shown in Fig. 5.

### Ablation study

To verify the importance of various parts of CDMENet, a series of ablation studies are conducted.

Fixed number of unlabeled images: To verify the effect of labeled images on CDMENet performance, the number of unlabeled images is fixed at 50% of the training set (fixed-unlabeled), and the number of labeled images ranged from 10% to 50% of the training set. The experimental results are shown in Table 2. It can be seen that CDMENet becomes more and more effective as the number of labeled images increases, which proves the robustness of the model. After reaching a certain number of labeled images (i.e., 20%), the gain in counting performance is no longer substantial. This further proves the full utilization of labeled and unlabeled images by CDMENet.

**Table 2.** Experimental results of fixed-unlabel images and label-only images on CDMENet

Ratio	Fixed-unlabeled			Label-only			CDMENet		
	MAE	RMSE	*R* ^2^	MAE	RMSE	*R* ^2^	MAE	RMSE	*R* ^2^
10%	55.13	80.49	0.9299	81.65	105.76	0.8832	30.57	40.21	0.9787
20%	30.73	41.85	0.9787	59.45	84.49	0.9183	28.01	37.65	0.9817
30%	28.49	38.95	0.9807	51.81	71.77	0.9366	27.58	37.22	0.9823
40%	27.61	36.50	0.9822	48.62	66.90	0.9455	27.12	36.12	0.9829
50%	26.47	36.32	0.9839	45.72	64.11	0.9496	26.47	36.32	0.9839

Using only labeled images on CDMENet: To verify the effect of unlabeled images on CDMENet performance, the inflow channel of unlabeled images is removed on CDMENet, and only labeled images are used to train CDMENet (i.e., Label-only). The experimental results are shown in Table 2. Compared to the results when unlabeled images are included, the results using labeled images only consistently show a decline in counting performance. This demonstrates the importance of unlabeled images for enhancing the performance of CDMENet.

The importance of density difference loss for CDMENet: Density difference loss enhances feature difference between density levels, fully leveraging the potential of unlabeled images. The experimental results of the importance of density difference loss for CDMENet are shown in Table 3. The counting performance decreases when density difference loss is not used, which proves the importance of density difference loss to CDMENet.

**Table 3.** The impact of density difference loss on the performance of CDMENet

Method	MAE	RMSE	*R* ^2^
CDMENet w/o $L_{\mathrm{DD}}$	48.37	69.07	0.9458
CDMENet	30.57	40.21	0.9787

Number of auxiliary task predictors: To verify the impact of the number of predictors on counting performance, the number of predictors is added and reduced. In particular, 2 and more predictors are required for the density mutual exclusion relation. The experimental results are shown in Table 4. The results show that the best counting performance can be obtained when the number of predictors is 3. The higher fine-grained density level classification does not necessarily lead to less counting errors.

**Table 4.** Experimental results of changing the number of predictors on CDMENet

Number	MAE	RMSE	*R* ^2^
2	33.67	43.85	0.9723
3	30.57	40.21	0.9787
4	31.02	41.26	0.9779
5	32.16	42.79	0.9756

Prediction confidence threshold: The prediction confidence *t_p_* is the threshold value for generating pseudo-class labels for predicted binary segmentation maps. ** The experimental results demonstrating the influence of changing *t_p_* on CDMENet are presented in Table 5. The change of *t_p_* has a minor impact on the performance of CDMENet, proving the stability and robustness of the proposed method.

**Table 5.** Experimental results of changing the prediction confidence threshold on CDMENet

Method	MAE	RMSE	*R* ^2^
*t_p_* = 0.7	30.62	40.34	0.9786
*t_p_* = 0.8	30.57	40.21	0.9787
*t_p_* = 0.9	30.64	40.41	0.9785

## Conclusion

To solve the grape berry counting problem cost-effectively, a semi-supervised counting of grape berries in the field based on density mutual exclusion is proposed. The method uses VGG16 as the backbone network. The auxiliary task based on density mutual exclusion are introduced, which reduce the interference of noise from unlabeled images to the density regressor, and the spatial information of labeled and unlabeled images is fully utilized to improve counting performance of the model. Finally, a density difference loss is proposed to expand the differences of features between different density levels. CDMENet is compared with fully supervised and semi-supervised counting algorithms on the field grape berry dataset. The experimental results show that CDMENet achieves less counting errors. Compared with the state of the arts, *R*^2^ is improved by 6.10%, and MAE and RMSE are reduced by 49.36% and 54.08%, respectively. CDMENet shows superior counting performance when the number of labeled images used are less. Extensive ablation studies also demonstrate the potential of CDMENet in reducing cost of labeling. In addition, density difference loss cannot completely eliminate false predictions. We may consider investigating more optimal losses to reduce errors in feature differences in future work and will explore the possibility of deploying the algorithm in this paper on the field robot or realistic platforms.

## Data Availability

The data presented in this study are available on Github (https://github.com/youth-tang/CDMENet-main).
